# Pluripotency of Stem Cells from Human Exfoliated Deciduous Teeth for Tissue Engineering

**DOI:** 10.1155/2016/5957806

**Published:** 2016-05-30

**Authors:** Vinicius Rosa, Nileshkumar Dubey, Intekhab Islam, Kyung-San Min, Jacques E. Nör

**Affiliations:** ^1^Oral Sciences, Faculty of Dentistry, National University of Singapore, Singapore 119083; ^2^Discipline of Oral and Maxillofacial Surgery, Faculty of Dentistry, National University of Singapore, Singapore 119083; ^3^Department of Conservative Dentistry, School of Dentistry, Chonbuk National University, Jeonju 54596, Republic of Korea; ^4^Department of Cariology, Restorative Sciences and Endodontics, School of Dentistry, University of Michigan, Ann Arbor, MI 48109, USA

## Abstract

Stem cells from human exfoliated deciduous teeth (SHED) are highly proliferative pluripotent cells that can be retrieved from primary teeth. Although SHED are isolated from the dental pulp, their differentiation potential is not limited to odontoblasts only. In fact, SHED can differentiate into several cell types including neurons, osteoblasts, adipocytes, and endothelial cells. The high plasticity makes SHED an interesting stem cell model for research in several biomedical areas. This review will discuss key findings about the characterization and differentiation of SHED into odontoblasts, neurons, and hormone secreting cells (e.g., hepatocytes and islet-like cell aggregates). The outcomes of the studies presented here support the multipotency of SHED and their potential to be used for tissue engineering-based therapies.

## 1. Introduction 

Dental pulp is a highly vascularized connective tissue encapsulated in mineralized structure formed by enamel, dentin, and cementum. It is responsible for the homeostasis of the tooth organ and acts as a sensor to detect unhealthy stimuli [1]. The dental pulp is a source of different populations of stem cells, such as dental pulp stem cells (DPSC) in permanent teeth and stem cells from human exfoliated deciduous teeth (SHED) [2]. The latter are isolated from primary teeth, which are one of the only naturally disposable and readily accessible postnatal human tissues [3]. In fact, SHED can be isolated even from carious deciduous teeth [4]. Moreover, there are very limited ethical or legal concerns about the use of these cells, hence increasing the interest in SHED for tissue engineering research [3].

SHED were first isolated in 2003 from exfoliated human deciduous incisors. The characterization of the cells obtained in that and subsequent studies showed that SHED present positive expression for a set of embryonic stem cell markers (OCT4 and NANOG), stage-specific embryonic antigens (SSEA-3 and SSEA-4), mesenchymal stem cell markers (STRO-1 and CD146), and tumor recognition antigens (TRA-1-60 and TRA-1-81) but negative for the expression of hematopoietic markers such as CD45, CD11b/c, and HLADR [5–8].

Deciduous and permanent teeth are considerably different in regard to their development, morphological features, and physiological processes. Consequently, it is expected that SHED differ from DPSC with respect to their higher proliferation rate, sphere-like cell-cluster formation, and differentiation capacity [5, 7, 9–14]. For instance, SHED present higher levels of osteocalcin production and alkaline phosphatase activity than DPSC during osteogenic differentiation [10]. Similarly, after neurogenic stimulation, SHED present higher expression of *β* III-tubulin, tyrosine hydroxylase, microtubule-associated protein 2, and nestin compared with the DPSC [15].

SHED can cross lineage boundaries and differentiate into several types of cells, such as adipocytes, endothelial cells, and neurons [7, 12]. SHED can also undergo osteogenic differentiation and generate bone* in vivo*, making these cells an interesting model for bone tissue regeneration [7, 16]. This has been highlighted by a study where SHED mixed with *β*-tricalcium phosphate carrier were able to promote bone regeneration in jaw defects in swines while the carrier without cells failed to induce the same [17]. Similarly, SHED mixed with platelet-rich plasma were able to promote the formation of vascularized mature bone in defects created in the mandible of the dogs after 8 weeks [18]. Altogether, these findings expand the potential of SHED to be used for tissue engineering-based therapies involving a large number of tissues.

## 2. SHED for Dental Pulp Tissue Engineering

Despite the introduction of new materials, medicines, and tools for the clinical management of dental pulp diseases, the principles of root canal treatment have not evolved significantly from the disinfection and obturation paradigm. This well-established approach presents high rates of success in the daily clinics, but it is merely based on the substitution of organic tissues with synthetic and, in many cases, inert materials [11]. This often restricts the completion of root development in immature teeth [19]. Hence, the development of clinically approachable techniques that allow the regeneration of a functional dental pulp that is capable to deposit organized and mineralized matrix is of great interest [3, 20]. The shift towards regenerative endodontics can lead to the rescue of tooth viability and further development of the root structure.

The first evidence that SHED could be used for dental pulp tissue regeneration was presented in the landmark paper published by Miura et al. in 2003. There, SHED were mixed with hydroxyapatite/tricalcium phosphate and implanted in the subcutaneous space of immunocompromised mice. After 8 weeks, SHED from either single or multiple colonies were capable to survive and proliferate within the scaffold and form dentin-like tissue [7].

The inherent potential of SHED to induce the formation of dental pulp* in vivo* gained momentum later in 2008. In that year, Cordeiro and collaborators seeded SHED in biodegradable poly-L-lactic acid-based scaffolds prepared within human tooth slices of 1 mm in thickness that were subsequently implanted in mice. After four weeks, a pulp-like tissue with a vascular network was formed in the space once occupied by the scaffold. In addition, cells lining the dentin surface morphologically resembled odontoblasts presenting eccentric polarized position of the nucleus on the basal part of the cell body and positive protein expression for dentin sialoprotein (DSP) [21].

The capacity of SHED to differentiate into fully functional odontoblasts capable of depositing a mineralized structure comparable to dentin* in vivo* was observed later. Similar to the previous study, SHED were seeded within a scaffold cast in a tooth slice and implanted subcutaneously into the dorsum of mice. 32 days after implantation, a dental pulp-like tissue was centripetally formed in the pulp chamber of the tooth slice. The tissue formed with SHED had a positive expression for markers of odontoblastic differentiation such as dentin sialophosphoprotein (DSPP) and dentin matrix protein 1 (DMP-1). During the experiment, mice received periodical injections of tetracycline hydrochloride to evince the deposition of mineralized matrix. Remarkably, well-defined fluorescent lines originated from the chelation of calcium ions in the newly deposited dentin offered the evidence that SHED can differentiate into fully functional odontoblasts* in vivo* [12].

**Figure 1:**
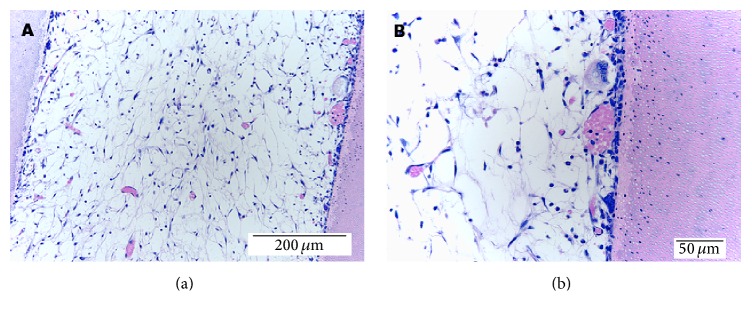
Dental pulp tissue engineered by the transplantation of SHED loaded in an injectable scaffold (a) and tooth extracted for orthodontic reasons (b). Reprinted with permission from [11] (Copyright (2011) John Wiley and Sons).

The translation of pulp tissue engineering to clinical setting requires the regeneration of the dental pulp within the full length of the root canal. SHED were shown to be capable to attach to the dentin walls and proliferate inside the root canals* in vitro* [22]. In 2013, SHED transplanted into full length root canals with injectable scaffolds were capable of proliferating within the root canal and express putative markers of odontoblastic differentiation (DSPP, DMP-1, and MEPE) after 28 days* in vitro*. In addition, there was the generation of a functional dental pulp in full length root canals* in vivo* when the roots with SHED were implanted in the subcutaneous space of mice (Figure 1). Remarkably, after 28 days from implantation, a human pulp-like tissue was occupying the majority of the space of the root canal regardless of the type of injectable scaffold used. The engineered dental pulp was capable of depositing new dentin as evinced by tetracycline injections at a rate of approximately 10 *μ*m/day [23]. The growth rate observed is within the range reported for primary dentin that varies according to the stage of development and age of the tooth (4 to 15 *μ*m/day) [24]. Though these findings are exciting, they are related to the ectopic tissue formation. Future studies must be performed to evaluate the capability of SHED to generate functional dental pulp in the oral environment.

## 3. SHED for Neuron Tissue Engineering

Neurodegenerative disorders are characterized by the loss or degeneration of neurons, leading to functional impairment. Although some of them, such as Parkinson and Alzheimer's disease, mainly affect older people, they are not part of the natural aging process. Parkinson disease affects 9.7 to 13.8 per 100,000 population, while Alzheimer's disease has become a major public health concern as the world's population ages [25, 26]. In addition to these diseases, there are approximately five million people living with traumatic brain disability in the United States alone [27]. Other injuries such as stroke, peripheral nerve injury, and spinal cord injury also pose a huge burden to society. Due to the limited regenerative capacity of the nervous system, stem cell-based therapies have emerged as therapeutic options. The neural crest-cell origin of the dental pulp makes SHED an interesting cell model for neuron tissue regeneration research [28].

SHED under nonneuronal induction conditions express nestin, glial fibrillary acidic protein (GFAP), doublecortin, neuronal nuclei (NeuN), and others at both the genetic and protein levels [7, 8, 29–31]. Although SHED present neuronal traits in their undifferentiated state, these cells are prone to undergo neurogenic differentiation both* in vitro* and* in vivo* [7, 32–34]. SHED treated with culture medium supplemented with epidermal growth factor (EGF) and fibroblast growth factor (FGF) presented high protein expression of neuronal markers including *β*III-tubulin, glutamic acid decarboxylase (GAD), and NeuN after four weeks. Nonetheless, there was no increase in the expression of nestin, GFAP, and neurofilament M (NFM) [7]. A similar trend was observed in another study where differentiated SHED showed positive expression of both glial and neuronal markers after 21 days. Here, some of the differentiated cells presented deposits of antimyelin basic protein while the majority of cells displayed positive expression for neuronal marker *β*III-tubulin. In addition, differentiated SHED presented a positive expression of apolipoprotein E (Apo E), which is present within the glia surrounding motor and sensory neurons in the peripheral nervous system. Differentiated cells were also positive for intermediate filament peripherin and Brn3a [32]. The first is a protein present in the peripheral nervous system while the latter is a transcription factor that regulates peripheral sensory neurons differentiation [32, 35].

The neural developmental potential of SHED* in vivo* was demonstrated by Miura and colleagues by injecting SHED into the dentate gyrus of the hippocampus of mice. The cells survived in the environment provided and continued to express neural markers such as NFM for more than ten days [7].

The potential of SHED to undergo neurogenic differentiation* in vivo* opened avenues for the use of these cells as an alternative model to treat different neuron-related conditions like focal cerebral ischemia, spinal cord injuries, Alzheimer's disease, and others.

Focal cerebral ischemia occurs when there is not enough oxygen supply due to limited blood flow to a specific region of the brain and it may lead to cerebral infarction or ischemic stroke [36, 37]. SHED can secrete compounds that positively influence the recovery of this type of lesion. Rats with focal cerebral ischemia induced by permanent middle cerebral artery occlusion presented with a significant decrease in the motor disability score when subjected to intranasal administration of supernatants from the medium used to culture SHED compared to Dulbecco's Modified Eagle's Medium (DMEM) or bone marrow stem cells conditioned DMEM used as controls. Furthermore, there was a significant decrease in the infarct volume from approximately 140 mm^3^ for DMEM to 50 mm^3^ when SHED conditioned medium was used. In addition, the animals treated with SHED conditioned media had more positive signals for neuronal nucleus, neurofilament H, doublecortin, and rat endothelial cell antigen in the peri-infarct area when compared with the DMEM used as a control [38].

**Figure 2:**
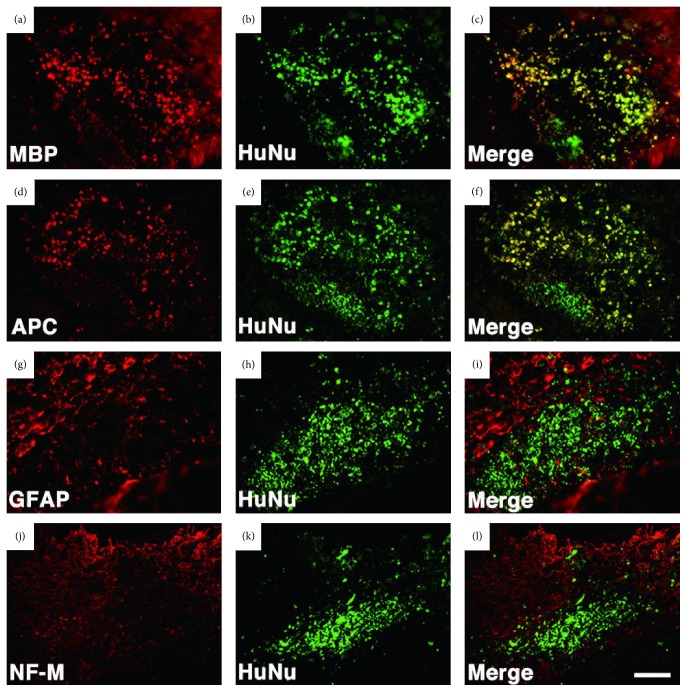
Immunohistochemical staining showed that SHED transplanted into transected spinal cord differentiated into mature oligodendrocytes [8] (Copyright (2012) American Society for Clinical Investigation.)

A spinal cord injury is a life-disrupting condition that triggers irreversible loss of motor and sensory functions [39]. It is estimated that up to 500,000 people suffer from spinal cord injuries per year worldwide and some severe types (e.g., C4 or higher lesions) may negatively affect breathing since the lesion affects the autonomic control system [40]. SHED have remarkable neuroregenerative activity and can promote functional recovery after spinal cord injury. Rats that received SHED within the space created by the complete transection at the 9th–11th thoracic vertebral levels exhibited higher scores in the Basso, Beattie, and Bresnahan locomotor rating scale compared to those transplanted with bone marrow stromal cells or fibroblasts. The rescue of hind limb locomotor function was also higher in the rats that received SHED. These animals were able to move three joints of hind limb coordinately and walk, while the treatment with bone marrow stromal cells allowed only subtle movements of one or two joints. The improvements observed with SHED can be related to their differentiation into mature oligodendrocytes that replace lost cells and regeneration of cleaved axons (Figure 2) [8].

The enhanced regeneration observed with SHED not only applies to lesions in the central nervous system, but also was observed in peripheral nerve injuries. It was possible to promote the regeneration of sciatic nerve defects in rats by treating the lesions with SHED conditioned media. The axon density and number of regenerated myelinated fibers observed in the group treated with SHED conditioned media were similar to the autograft used as a control [41].

These promising results pertaining to the neuron differentiation potential of SHED in several* in vitro* settings and animal models increase the interest of using these cells as an alternative for treating different neuronal diseases and injuries.

## 4. SHED Differentiation towards Hormone Secreting Cells

In addition to their therapeutic use in dental pulp and neuroregeneration, SHED also have the potential to be used in the treatment of hepatic diseases and diabetes.

Organ transplantation may be the choice of treatment for patients suffering from fatal liver conditions such as cirrhosis and hepatocellular carcinoma. However, the scarcity of donors encourages the development of therapeutic alternatives. Recent studies provide evidence that SHED can differentiate into hepatic lineage cells [42–45]. Under proper stimulation, SHED express a set of hepatic markers such as hepatic nuclear factor-4*α*, *α*-fetoprotein, and insulin-like growth factor-1. Remarkably, 90% of the hepatocytes obtained were positive for the expression of albumin. In addition, there were significant increases in the concentration of urea in the media and amount of cytoplasmic glycogen storage within the cells after differentiation [43]. The level of differentiation can be further enhanced by addition of liquorice or angelica extracts in the culture medium [46] or by treating with hydrogen sulfide (H_2_S) [47]. The transplantation of SHED into the liver of mice with fibrosis induced by carbon tetrachloride showed that the cells can participate in the hepatic recovery via both direct (tissue replacement) and indirect (antifibrotic and anti-inflammatory effects) integration [48].

Diabetes is one of the most common chronic endocrinal diseases associated with destruction and dysfunction of pancreatic *β*-cells. As SHED can differentiate* in vitro* into islet-like cell aggregates (ICA), they emerge as an alternative towards cell replacement therapy for diabetes [49, 50]. It has been shown that the SHED-originated ICA can release insulin and C-peptide in a glucose-dependent manner* in vitro* [49]. The incubation of MIN6 (mouse pancreatic *β*-cell line) with SHED conditioned medium enhanced insulin secretion in a glucose concentration-dependent manner [51]. Mice with diabetes induced by streptozotocin which were transplanted with islet-like cells derived from SHED reversed the diabetes and restored the normoglycemia after three to four weeks [50].

## 5. Conclusion

As SHED can be obtained from naturally “disposable” tissues without significant morbidity to host and with limited ethical concern, they present another opportunity for dentistry to contribute to the development of tissue engineering. Several studies offer the evidence that SHED can differentiate into odontoblasts, neurons, hepatocytes, endothelial cells, *β*-cells, and others. This wide variety of cell types creates a plethora of opportunities for the use of SHED in tissue regeneration processes. Nonetheless, there is still the need to deepen the understanding of the mechanisms underlying the differentiation processes before SHED-based therapies can become a clinical reality.
